# CCL5 Levels Predict Stroke Volume Growth in Acute Ischemic Stroke and Significantly Diminish in Hemorrhagic Stroke Patients

**DOI:** 10.3390/ijms23179967

**Published:** 2022-09-01

**Authors:** Francisco José Julián-Villaverde, Marta Serrano-Ponz, Enrique Ramalle-Gómara, Alfredo Martínez, Laura Ochoa-Callejero

**Affiliations:** 1 Stroke Unit, Neurology Service, Hospital San Pedro, 26006 Logroño, Spain; 2 Neurology Service, Hospital Universitario Miguel Servet, 50009 Zaragoza, Spain; 3 Department of Epidemiology, La Rioja Government, 26071 Logroño, Spain; 4 Angiogenesis Group, Oncology Area, Center for Biomedical Research of La Rioja (CIBIR), 26006 Logroño, Spain; 5 Department of Nursing, University of La Rioja, 26004 Logroño, Spain

**Keywords:** CCL5, ischemic stroke, hemorrhagic stroke, temporal profiles, stroke volume growth

## Abstract

Stroke remains an important health challenge. Here, we study whether circulating chemokine (C-C motif) ligand 5 (CCL5) levels may predict clinical outcomes for stroke patients. A total of 100 consecutive stroke patients (36 acute ischemic and 64 hemorrhagic) were admitted to the stroke unit. Clinical history data and monitoring parameters were recorded. Blood serum was collected at days 0, 1, and hospital discharge to measure CCL5 levels by ELISA. Infarct or hemorrhagic volume, neurological severity (NIHSS), and functional prognosis (mRankin scale) were measured as clinical outcomes. CCL5 levels were lower in patients with hemorrhagic stroke than in patients with acute ischemic stroke. No differences were found between females and males in both types of stroke. Ischemic stroke patients whose infarct volume grew had lower CCL5 levels at day 0. Levels of CCL5 in ischemic and hemorrhagic patients were not associated with more severe symptoms/worse prognosis (NIHSS > 3; mRankin > 2) at admission or at 3 months. CCL5 could be used as a diagnostic marker to distinguish between ischemic and hemorrhagic strokes. Furthermore, CCL5 levels could predict the infarct volume outcomes in ischemic patients.

## 1. Introduction

Stroke is the second leading cause of death in the world and the most common cause of disability in elderly people worldwide [1]. The World Health Organization estimates that one in six people will suffer from stroke in their lifetime [2]. The annual incidence of stroke is approximately 0.2% in the general population. Approximately 15 million people suffer from stroke every year, and of these, 5 million will die while other 5 million will remain permanently incapacitated [3].

A stroke occurs when an area of the brain is suddenly deprived of blood flow. This may be due to the occlusion of a blood vessel (ischemic stroke) or to a local intracranial hemorrhage (hemorrhagic stroke). Approximately 80% of all strokes are ischemic, most commonly caused by a cardiogenic embolism, cerebral microcirculatory impairment, atherosclerosis of extracranial or intracranial arteries, or blood clotting disorders [4,5,6]. Oxidative stress and inflammatory reaction constitute the key mechanisms leading to neural damage. The lack of nutrients and oxygen alters the metabolism of the affected neurons and glial cells, and results in the appearance of neurological symptoms and signs that could become irreversible, depending on the length of time until circulation is re-established [7,8]. Current data suggest that up to 85% of all strokes may be preventable through medical intervention and lifestyle modifications [9], but hospitals still receive many patients in stroke units needing urgent care.

Over the past few years, a search for the biomarkers that could predict clinical outcomes in stroke patients has been ongoing [10]. Acute inflammation after stroke is a multifaceted response to sterile tissue injury. Inflammation is involved in triggering mechanisms that eventually promote clearance of the damaged tissue and set an adequate environment for subsequent tissue repair, although excessive inflammation could exacerbate the brain lesions [11]. As part of the initial phase of inflammation, diverse types of leukocytes are attracted to the injured brain areas in an orchestrated fashion, where they carry out complex functions [12,13]. Leukocyte attraction is regulated by pro-inflammatory mediators (such as cytokines, chemokines, adhesion molecules, and matrix metalloproteinases) [14]. Chemokines can be released by neurons, astrocytes, microglial cells, oligodendrocytes, leukocytes, and endothelial cells [15,16]. Interestingly, a dual role has been hypothesized for inflammation and chemokines, which could be either potentially deleterious or restorative, thus making this pathway an interesting target for therapeutical modulation [17].

Chemokine (C-C motif) ligand 5 (CCL5) is a chemokine whose involvement in stroke is attracting interest [14,18,19,20,21,22]. CCL5 is a member of the CC chemokine family that is produced by a variety of cells, including T lymphocytes, platelets, endothelial cells, smooth muscle cells, and glial cells. It can interact with chemokine receptors that recruit leukocytes to inflammatory sites, assisting with their migration across the endothelium and contributing to the pathogenic process of arterial injury and atherosclerosis [23,24]. T-cell recruitment and activation in the injured brain parenchyma could be the prelude to secondary ischemic injury [25,26]. Many studies have shown significant correlations between CCL5 levels and atherosclerotic plaque progression, cardiac injury, and markers of heart failure, even among patients with acute coronary syndromes complicated by sudden cardiac arrest [27].

CCR5 is the main receptor for CCL5, although this chemokine can also bind with varying affinity to CCR1 and CCR3 [28]. CCR5 is expressed in macrophages, activated T-cells, natural killer cells, endothelial cells, and endothelial progenitor cells (EPCs), among others [29]. CCR5 participates in the regulation of the pro-inflammatory response by modulating the behavior, survival, and retention of immune cells in tissues [30]. In addition, CCR5 can be expressed in non-immune cells, notably in astrocytes and in neurons, where it is involved in neuronal survival and differentiation [31].

For our purpose, it is important to note that CCR5 is specifically expressed in endothelial cells and EPCs. CCR5 facilitates progenitor cell recruitment and promotes vascular endothelial repair during the process of endothelial damage [32,33,34]. Although inhibiting CCR5 expression reduces the inflammatory response, it also aggravates endothelial damage, thus significantly limiting the actual effectiveness of CCR5 inhibitor-based therapeutic interventions [29].

Several publications have studied the levels of circulating CCL5 in ischemic stroke patients; however, there is some controversy, as several of them reported higher levels of CCL5 in stroke compared with healthy controls [18,19,21], while others found no differences [14,20,22]. To the best of our knowledge, there is no published information on the levels of CCL5 in hemorrhagic stroke.

Taking all this into consideration, we elected to perform a longitudinal follow-up of stroke patients, both ischemic and hemorrhagic, measuring their circulating levels of CCL5 at different times after stroke onset, and testing whether these values may be predictive of clinical parameters such as infarct volume growth, neurological severity, and functional prognosis.

## 2. Results

The clinical sample included both ischemic and hemorrhagic stroke patients. The first group comprised 36 acute ischemic stroke cases, 16 women (44.5%) and 20 men (55.5%), with a median age of 75 years (Table 1). Some patients had been exposed to relevant risk factors such as arterial hypertension, diabetes, dyslipidemia, atrial fibrillation, or a previous stroke (Table 1). An important proportion of these patients had been treated with antihypertensives, statins, antiaggregants, or anticoagulants (Table 1). After completing an etiologic profile, 30.5% of patients were diagnosed with atherothrombotic stroke, 44.4% with cardioembolic stroke, 5.5% with lacunar stroke, 13.8% with cryptogenic forms of stroke, and 5.5% with other forms of stroke (Table 1). The median National Institutes of Health Stroke Scale (NIHSS) taken at admission was 6.0, with a median infarct volume of 4.9 cm^3^ (Table 1). Large vessel occlusion was demonstrated in 9 patients (25%) out of 36.

The second group included 64 hemorrhagic stroke patients, 26 women (40.6%) and 38 men (59.4%), with a median age of 81 years (Table 2). The risk factors and previous treatments were similar to those of the ischemic group (Table 2). After completing the etiologic profile, more than half of the patients (64.1%) were diagnosed with supratentorial stroke, 21.9% with lobar stroke, 9.4% with infratentorial stroke, and 4.7% with mixed stroke (Table 2). The median NIHSS taken at admission was 7, with a median hematoma size of 4.5 cm^3^ (Table 2).

### 2.1. CCL5 Levels Are Lower in Stroke Patients Than in Healthy Controls

**Figure 1:**
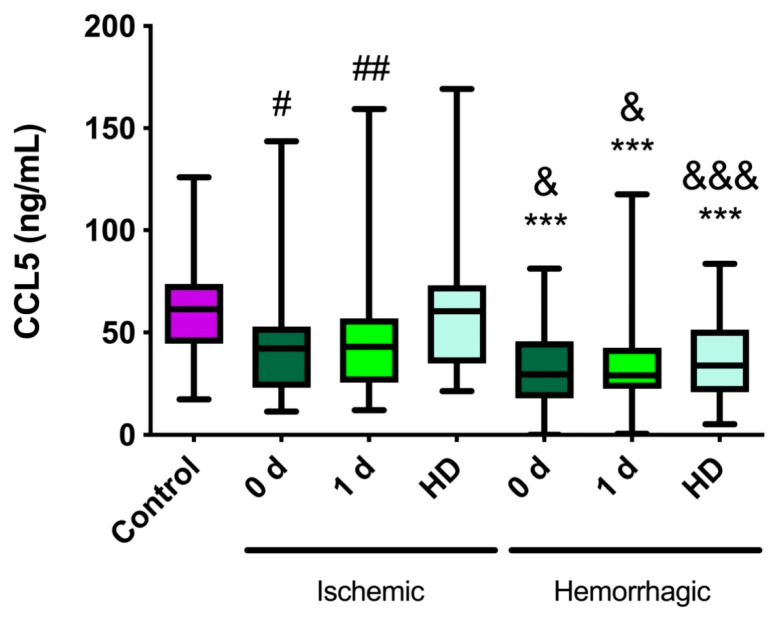
**Evolution of CCL5 levels in acute ischemic and in hemorrhagic stroke patients.** CCL5 was measured in healthy controls (n = 31), ischemic stroke patients (n = 36), and hemorrhagic stroke patients (n = 64), either at admission (0 d), the following day (1 d), or at hospital discharge (HD). Ischemic patients exhibited no change in CCL5 levels compared with controls. Hemorrhagic patients had lower CCL5 levels at every time point compared with healthy volunteers. Furthermore, hemorrhagic stroke patients had lower CCL5 levels than ischemic stroke patients at every time point. Box plots represent the interquartile range with the median as the horizontal line. Whiskers encompass the maximum and minimum values of the population. ***: *p* < 0.001 vs. control; ^#^: *p* < 0.05; ^##^: *p* < 0.01 vs. HD ^&^: *p* < 0.05; ^&&&^: *p* < 0.001 between stroke types at the same time point.

In healthy control subjects, the median (Q1–Q3) CCL5 level was 61.2 ng/mL (46.1–73.4) (Figure 1). No significant differences were observed between the sexes (*p* = 0.279). In the ischemic stroke patients, no significant differences were found compared with healthy controls. Nevertheless, at hospital discharge (HD), the CCL5 levels were significantly higher than at day 0 (*p* = 0.028) and at day 1 (*p* = 0.007) (Figure 1). In hemorrhagic stroke patients, the CCL5 levels measured at every time point were significantly lower than those obtained in healthy subjects (*p* ≤ 0.001), with no significant differences among times (Figure 1).

### 2.2. CCL5 Levels Are Lower in Hemorrhagic Stroke Patients Than in Ischemic Stroke Patients

Interestingly, patients with hemorrhagic stroke had lower CCL5 levels than patients with ischemic stroke at every time point (1.41-fold: 30.4%, 1.48-fold: 32.8%, and 1.77-fold: 43.7%, 0-1-HD, respectively; *p* = 0.014, *p* = 0.028, and *p* < 0.001, respectively) (Figure 1).

### 2.3. CCL5 Levels and Their Relationship with Sex

**Figure 2:**
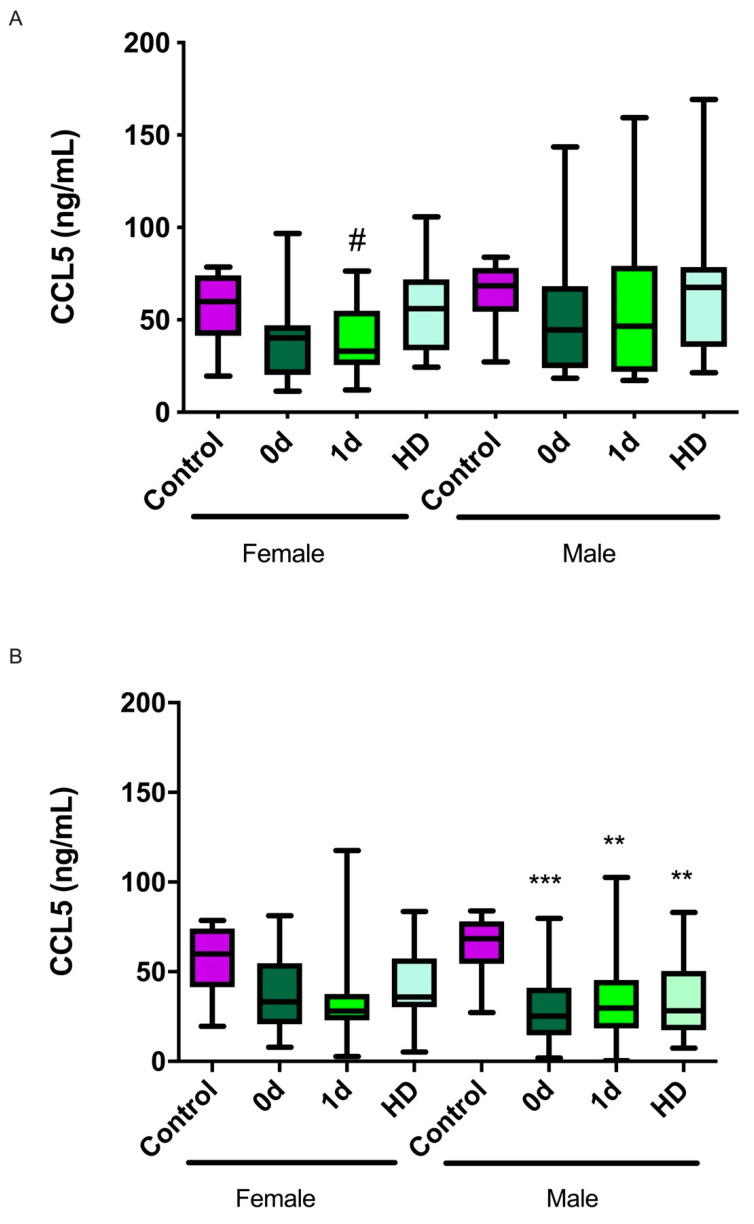
**Association between CCL5 levels and sex.** (**A**) Female ischemic stroke patients (n = 16) showed lower CCL5 levels at 1 d than at HD, whereas males (n = 20) showed no differences. (**B**) In hemorrhagic stroke patients, males (n = 38) showed significantly lower levels than controls. Box plots represent the interquartile range with the median as the horizontal line. Whiskers encompass the maximum and minimum values of the population. **: *p* < 0.01; ***: *p* < 0.001; vs. control; ^#^: *p* < 0.05 vs. HD.

When the levels of CCL5 were categorized by sex, we observed that in the ischemic patients, significant differences were found only in women between day 1 and HD (*p* = 0.01), whereas no significant changes were detected among men (Figure 2A). On the other hand, in the hemorrhagic stroke patients, the CCL5 levels were significantly lower than those in the healthy subjects only in the male group (*p* < 0.001 at day 0, *p* = 0.003 at day 1, and *p* = 0.001 at HD) (Figure 2B).

### 2.4. CCL5 Levels Are Lower in Cardioembolic and Lacunar Subtypes of Ischemic Stroke

According to the Trial of ORG 10172 in Acute Stroke Treatment (TOAST) classification, five subtypes of ischemic stroke are present among our patients: atherothrombotic (n = 11), cardioembolic (n = 16), lacunar (n = 2), cryptogenic (n = 5), and undetermined etiology cause (n = 2). CCL5 levels significantly decreased in the cardioembolic and lacunar subtypes at admission and at day 1, whereas they did not change significantly in the other subtypes (Supplementary Figure S1).

In addition, four TOAST subtypes of hemorrhagic stroke were found: supratentorial (n = 41), infratentorial (n = 6), lobar (n = 14), and mixed etiology (n = 3). Most of the subtypes, other than the infratentorial, had significantly lower levels of CCL5 at some time points (Supplementary Figure S2).

### 2.5. CCL5 Levels and Their Association with Infarct Volume or Hematoma Size Growth

**Figure 3:**
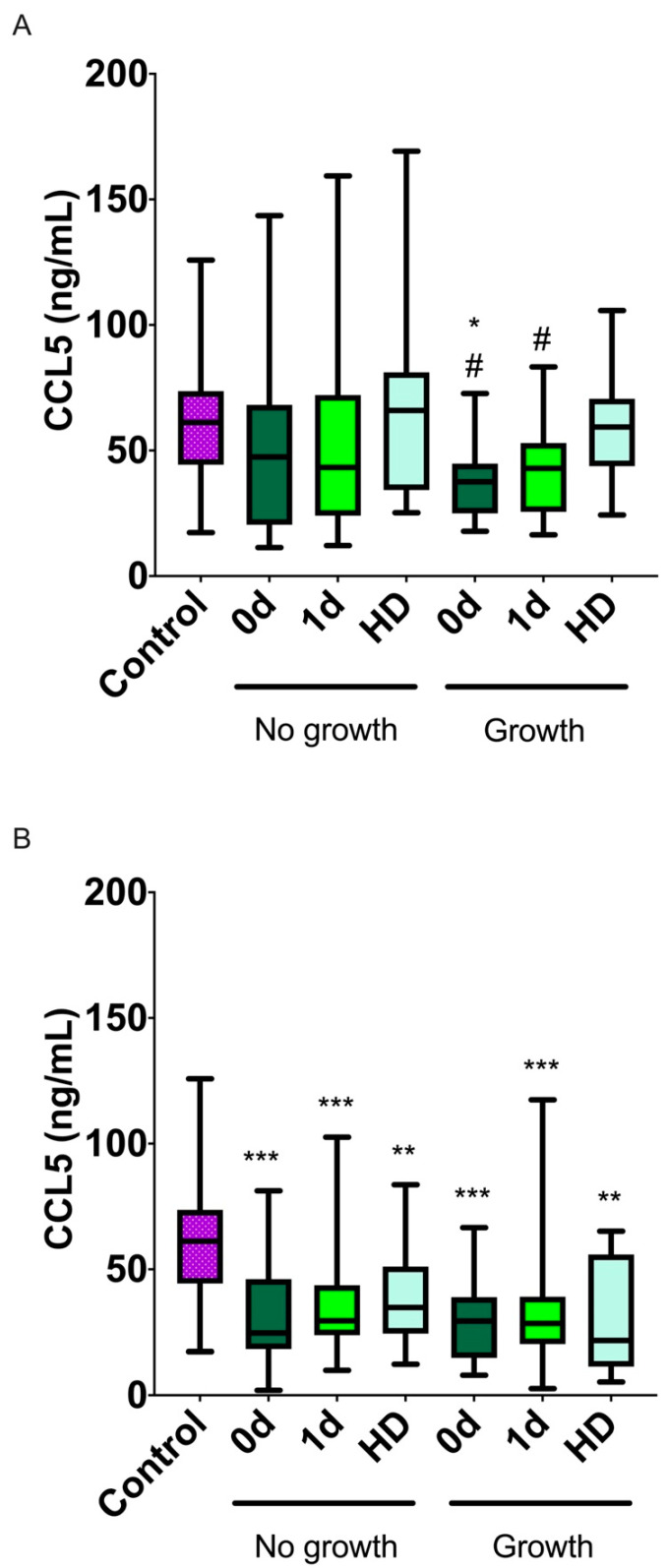
**CCL5 levels and growth in infarct volume (A) or in hematoma size (B).** CCL5 was measured in ischemic (**A**) and hemorrhagic (**B**) stroke patients whose infarct volume or hematoma size had grown (≥30%) or not (<30%). Ischemic patients whose infarct volume grew had lower CCL5 than patients whose infarct volume did not grow at day 0. Moreover, CCL5 levels at day 0 and day 1 were lower than at HD. No differences were found in hemorrhagic stroke patients. Box plots represent the interquartile range with the median as the horizontal line. Whiskers encompass the maximum and minimum values of the population. *: *p* < 0.05; **: *p* < 0.01, ***: *p* < 0.001 vs. control; ^#^: *p* < 0.05 vs. HD.

In the ischemic patients, infarct volume growth was defined as the difference between the infarct volume measured by nuclear magnetic resonance (NMR) at day 7 and the initial volume measured on admission. For 2 of the 34 ischemic patients, measuring infarct volume at day 7 was not possible because they died; therefore, the number of experimental data was reduced (n = 32). Patients were classified as having infarct volume growth when they had at least a 30% increase. A total of 15 patients (46.8%) experienced such growth, while 17 (53.1%) did not (Figure 3A). Ischemic patients with positive infarct volume growth had significantly lower CCL5 levels at 0 d compared with healthy volunteers (*p* = 0.04), and the CCL5 levels increased back to normal at HD (*p* = 0.012 and *p* = 0.025, compared with day 0 and day 1, respectively).

In the hemorrhagic patients, hematoma volume growth was defined as the difference between the hematoma volume measured by NMR at day 1 and the initial volume measured at admission. A total of 6 of 64 hemorrhagic patients died before the second measurement took place; therefore, the number of available data was reduced (n = 58). Patients were classified as having hematoma volume growth when they had at least a 30% increase. Hematoma growth was experienced by 20 patients (34.5%), while 38 patients (65.5%) had no growth (Table 2). All hemorrhagic patients showed significantly lower CCL5 levels at every time point in comparison with controls (healthy volunteers) (*p* ≤ 0.002). No differences were observed between the groups at any time point (Figure 3B).

### 2.6. CCL5 Levels and Their Relationship with NIHSS at Admission

The stroke patients were categorized as having a good (NIHSS ≤ 3) or bad (NIHSS > 3) prognosis at admission. In the good prognosis group of ischemic stroke patients, the CCL5 levels were significantly lower (*p* = 0.023) at day 1 than at HD (Supplementary Figure S3A).

In the hemorrhagic stroke patients, neurological severity at admission had no influence on CCL5 levels. Furthermore, in all hemorrhagic patients, the CCL5 levels were significantly lower than those of healthy controls (*p* ≤ 0.003) (Supplementary Figure S3B).

### 2.7. CCL5 Levels and Their Relationship with Neurological Severity at 3 Months

We should note that 4/23 (ischemic) and 22/42 (hemorrhagic) patients with a very high initial NIHSS score (≥5) died in the 3 months following their stroke.

The stroke patients were categorized as having a good (NIHSS ≤ 3) or bad (NIHSS > 3) prognosis at 3 months. In the bad prognosis group of ischemic stroke patients, the CCL5 levels were significantly lower (*p* = 0.008) at day 1 than at HD (Supplementary Figure S4A).

In the hemorrhagic stroke patients, no differences were found between prognosis groups (Supplementary Figure S4B), probably due to the low number of patients. Again, in all hemorrhagic patients, the CCL5 levels were significantly lower than those of healthy controls (*p* ≤ 0.003) (Supplementary Figure S4B).

Furthermore, we also analyzed the results for mRankin at 3 months. The stroke patients were categorized as having a good (mRankin ≤ 2) or bad (mRankin > 2) prognosis at 3 months. No differences were found between prognosis groups in either ischemic or hemorrhagic patients (data not shown).

## 3. Discussion

In this study, we found that patients suffering from either ischemic or hemorrhagic stroke had very different levels of CCL5. While these levels did not change in ischemic stroke compared with healthy individuals, there was a large reduction in CCL5 levels in the hemorrhagic stroke patients that lasted for at least 7 days post-stroke. This clear difference points to CCL5 as a potential diagnostic biomarker to differentiate both stroke manifestations. Despite the lack of change in CCL5 levels in ischemic stroke patients compared with healthy volunteers, we found a significant decrease, at admission, in patients whose stroke volume would grow, thus predicting a poorer outcome. Therefore, we can affirm that CCL5 levels at admission can be used as a prognostic biomarker to predict ischemic stroke patient evolution. On the other hand, no association was found between CCL5 levels and patient outcome in hemorrhagic stroke.

CCL5 is an intriguing chemokine that is produced from neural cells after ischemic stroke and has the potential to protect neurons directly or indirectly through the production of neurotrophic factors in peri-infarct areas [21]. Regarding whether circulating CCL5 levels climb or fall following ischemic stroke, there are different reports with contradictory data. Some studies described elevated levels with respect to healthy controls [18,19,21], while others found no differences in CCL5 levels compared with healthy controls or within ischemic stroke patients over time [14,20,22]. Moreover, in some of these studies [16,21], there was no control group to establish basal levels. These discrepancies may be due to the method of collecting samples at different times, the influence of patient conditions (sex, age, ethnicity), the type of stroke, or the stroke evolution over time. Other causes of variability may be found in pre-existing conditions, such as atherosclerosis; indeed, CCL5 was found to be involved in atherosclerotic lesion formation [35]. In addition, this variability may be associated with different extents and timing of reperfusion [36,37], and the different processes influencing post-stroke inflammation [38,39], including chemokine expression [40]. Apart from distinct study populations, different methods for testing CCL5 levels could also have contributed to the discrepancy in the above-mentioned studies. Our study reinforces the view that CCL5 levels do not change significantly in ischemic stroke, although there is a certain trend towards lower levels, especially among females, and the presence of lower levels of CCL5 at admission predicts stroke volume growth.

CCL5 has been described as a double-edged sword [29], thus making theoretical prediction difficult. This dual behavior may be based on the fact that inflammation triggers subsequent processes involved in cell migration, proliferation, matrix deposition, and tissue remodeling, which are important for tissue regeneration after stroke [41]. On the other hand, excessive inflammation could be a detrimental response that exacerbates brain damage after stroke [42]. An elevation in CCL5 levels could be neuroprotective as it would induce vasodilatation, an inhibition of platelet aggregation, and an induction of angiogenesis. Interestingly, Badacz et al. [18] described a higher mortality rate in patients with lower CCL5 levels due to cardiovascular complications. On the other hand, CCL5-knockout mice presented a decreased volume of ischemic area and significantly limited blood–brain barrier permeability, along with reduced leukocyte and platelet adhesion [43]. Furthermore, injecting a CCL5 inhibitor in experimental stroke animals reduced infarct sizes and improved neurological scores after stroke [44]. This detrimental effect of CCL5 may be mediated by its modulatory action on the immune system, reactivating other strong pro-inflammatory cytokines, which are important for stroke resolution [43]. In fact, the overexpression of CCL5 has been detected in the leukocytes of stroke patients, and this higher expression is associated with stroke severity [22]. Our observation that patients with lower levels of CCL5 at admission develop larger stroke volumes, and therefore have a worse prognosis, indicates that in ischemic stroke, CCL5 acts as a neuroprotective factor.

Our clinical observations agree with the data obtained from animal experiments, and suggest that during human stroke, CCL5 levels may be neuroprotective and may predict stroke recovery. For instance, Sorce et al. [31] showed that CCR5-deficient mice subjected to ischemic stroke showed a larger cerebral infarct size, with increased neuronal death and neutrophil infiltration compared with wild-type animals. Moreover, Ping et al. [45] revealed larger infarction sizes in CCR5^−/−^ mice in comparison with wild-type mice after experimental stroke. These studies suggest a potential neuroprotective role of CCR5 in ischemic stroke. However, the removal of the CCR5 receptor gene has a protective effect against cerebral ischemia and reperfusion injury [46,47].

Our results indicate that CCL5 downregulation may predispose patients to poor stroke outcome. This is in agreement with some of the literature [22]. In contrast, previous studies found elevated CCL5 content in the blood of ischemic stroke patients may be predictive of 3-month mortality and unfavorable outcomes, pointing to CCL5 as a negative predictor of clinical performance [18,19,20,21]. CCL5 may be able to play a neuroprotective role via CCR5 expressed in neurons in the early phase of stroke, while it may worsen neuronal damage by breaking the blood–brain barrier and inducing invasion by inflammatory cells through CCR1 expressed in vascular cells and white blood cells [21]. Therefore, the selective activation and/or inhibition of chemokine receptors at specific times may be critical when designing therapeutic drugs for ischemic stroke.

Interestingly, we found a non-significant trend, which may become significant in larger trials, suggesting a larger decrease in CCL5 levels in ischemic stroke female patients than in their male counterparts. This may correlate with epidemiological studies describing that women have a worse prognosis than men when exposed to ischemic stroke [48]. This has also been shown in experimental animals where the females presented larger stroke volumes than the males subjected to the same procedure [49]. Surprisingly, in hemorrhagic stroke, the decrease in CCL5 levels is more pronounced in males than in females. In agreement with our findings, clear sexual differences have also been described in hemorrhagic stroke patients: men showed higher perihemorrhagic edema after intracerebral hemorrhage [50], along with higher incidence, susceptibility, and mortality [51]. Moreover, unfavorable outcomes [52] and deeper hematomas occurred in men [53]. In contrast, other studies have suggested a higher stroke severity in women [54].

CCL5 levels were much lower in hemorrhagic stroke patients than in ischemic stroke patients. Therefore, CCL5 could be used as a diagnostic marker to distinguish between ischemic and hemorrhagic strokes. Along these lines, recent studies provide evidence that blood biomarkers, including retinol binding protein 4 (RBP-4), N-terminal prohormone of brain natriuretic peptide (NT-proBNP), glial fibrillary acidic protein (GFAP) [55], and adrenomedullin [56], differentiate between ischemic and hemorrhagic strokes with moderate accuracy.

The behavior of CCL5 in hemorrhagic stroke patients is presented here, for the first time, showing a significant reduction in level compared with that in healthy subjects. One possible explanation for this phenomenon may be the migration of chemokine-producing leukocytes into the interior of the brain, thus decreasing their levels in peripheral blood [57]. In addition, if CCL5 levels are neuroprotective, as discussed above, the lower levels of CCL5 in hemorrhagic stroke compared with those in ischemic stroke are in agreement with the higher clinical severity of hemorrhagic stroke.

In conclusion, CCL5 may act as a diagnostic biomarker differentiating between ischemic and hemorrhagic stroke. Furthermore, we have shown that CCL5 levels at admission predict the clinical outcome in ischemic stroke patients, as measured by infarct volume growth at day 7. Therefore, studying the mechanisms of CCL5/CCR5 biology that control endothelial cells and the inflammatory response will provide further understanding of the pathophysiology of cardiovascular disease, including stroke, and may assist with developing novel pharmacological strategies. Such findings provide further understanding of the pathophysiology of cardiovascular diseases, including CCL5 as a neuroprotective chemokine in stroke, and may be used to develop novel pharmacological strategies.

## 4. Material and Methods

### 4.1. Patients

This study was designed as a prospective, observational, and longitudinal clinical study of patients diagnosed with acute ischemic stroke at the Neurology Service of the Hospital San Pedro (Logroño, Spain) from October 2014 to April 2015, and of patients diagnosed with hemorrhagic stroke from December 2018 to January 2020. A total of 36 ischemic and 64 hemorrhagic consecutive stroke patients fulfilling the inclusion criteria signed the informed consent documents and were recruited into the study. The inclusion criteria for ischemic stroke called for patients suffering from acute ischemic stroke, as demonstrated by nuclear magnetic resonance (NMR), and an evolution of less than 24 h. The exclusion criteria were the same as those used for the hospital’s stroke unit and encompassed contraindications for performing NMR, age lower than 18 years, dementia, previous stroke within 3 months, cranial traumatism, infection in the central nervous system, cardiac insufficiency, renal failure, sepsis, active neoplasia, active inflammatory or autoimmune disease, pregnant or lactating women, and patients whose characteristics would prevent proper follow-up.

The inclusion criteria for hemorrhagic stroke were patients with intracerebral hemorrhage, as demonstrated on computer tomography scan (CT), with less than 6 h from onset of symptoms. The exclusion criteria were isolated subarachnoid hemorrhage, traumatic intracranial hemorrhage, patients fulfilling organ donation protocol, and neurosurgical hemorrhage.

Power calculation was performed using Granmo software v7.12 (Marrugat, J., Vila, J. Institut Municipal d’Investigación Médica, Barcelona: Antaviana; abril 1 April 2012. https://www.imim.es/ofertadeserveis/software-public/granmo/). Accepting an alpha risk of 0.05 in a two-sided test with 34 subjects in the first group (ischemic) and 64 in the second (hemorrhagic), the statistical power was 79% to recognize a difference of means as statistically significant: CCL5 levels of 46 ng/mL in group 1 and 32 ng/mL in group 2 at day 0.

### 4.2. Variables of the Study

The patients received standard care following the approved protocols of the stroke unit. The general characteristics of the patients were collected as part of the clinical history (age, sex, risk factors, current medical treatment, previous functional situation, etc.). During their stay at the stroke unit, several parameters were continuously monitored (electrocardiogram, systolic and diastolic blood pressure, temperature, and hypoxemia). Neurological severity was measured with the NIHSS scale [58] at day 0, day 1, HD, and 3 months. Functional prognosis was also evaluated with the mRankin scale [59] at 3 months. In addition, blood serum samples were taken after admission (day 0), at day 1, and at HD to quantify the circulating levels of CCL5. All ischemic stroke patients and most of the hemorrhagic patients were discharged at day 7, but some hemorrhagic patients were discharged between days 3 and 5 (n = 11), due to medical reasons. Ischemic stroke volume evolution was established by comparing the images taken using NMR on the first day with those taken at day 7. Infarct size was calculated on DWI at baseline and on FLAIR sequence at day 7. Infarct growth was defined as the difference between the day 7 FLAIR infarct volume and the baseline DWI lesion volume. Volumetric software (Advantage Windows 4.6, AW server 2-0.12, GE Medical Systems, Chicago, IL, USA) was used for infarct size (cc) measurements. The software analyses were based on the delineated infarct area and thickness of each slice. The perimeter of infarct was depicted by a unique blinded radiologist using a freehand technique. NMR was performed with a 3 Tesla instrument (Discovery MR 750w, GE Medical Systems, Milwaukee, WI, USA). Hemorrhagic stroke volume evolution was measured by comparing NMR at admission and at day 1. For intracranial hemorrhage, we used the ABC/2 formula to assess the volume of intracerebral hemorrhage. This formula has been well validated in several studies, and its measures are (1) hemorrhage shape, (2) hemorrhage length, (3) hemorrhage width, (4) number of CT slices with hemorrhage, and (5) CT slice thickness.

### 4.3. Determination of CCL5 Levels

The concentrations of CCL5 found in blood serum were determined using a commercially available ELISA kit (Cat. n#DRN00B, R&D systems, Minneapolis, MN, USA) following the manufacturer’s instructions. Samples were initially diluted 1/100, and CCL5 concentration was calculated by interpolation into a standard curve. CCL5 values are expressed as ng/mL. Intra-assay and inter-assay precision are coefficient of variation (CV) < 2.4% and CV < 6.5%, respectively.

### 4.4. Statistical Analysis

All statistical analyses were performed using the SPSS v.26 software package (IBM Corp. Armonk, NY, USA). First, a descriptive analysis of all variables was performed. Categorical variables were expressed as absolute and relative frequencies. Continuous variables were defined by their mean and standard error of the mean (SEM) when their distribution was normal (as tested by the Shapiro–Wilk test), or as the median and interquartile range when the distribution was not normal. Continuous normal variables were analyzed with a Student´s *t*-test or ANOVA. Repeated measures of ANOVA were used to test the effect of time on CCL5 values (0 days, 1 day, HD). When the samples did not follow a normal distribution, non-parametric tests, such as the Kruskal–Wallis test followed by the Mann–Whitney *U* test, were performed. Two-tailed tests were used and *p*-values < 0.05 were considered statistically significant.

## 5. Limitations

This study was a single-center observational (cross-sectional) study with a relatively small number of enrolled subjects. Multicentric and larger studies will be needed to corroborate our results. Commercially available assays measure total CCL5 protein, although variant forms, including those measuring truncated proteins and proteins with different post-translational modifications such as oxidation, glycation, or glycosylation, exist [18]. Furthermore, the CCL5 gene has several polymorphisms [60], some of which are associated with the risk of atherothrombotic cerebral infarction [24]. Identifying the specific contributions of all these variants to the outcome of stroke patients would be of interest. Furthermore, it has been shown that circulating CCL5 levels vary with race and with other patient characteristics [61]; therefore, we must be cautious when trying to extrapolate our findings to other populations.

## 6. Conclusions

In summary, ischemic stroke patients did not show different levels of CCL5 compared with healthy controls. Moreover, CCL5 may act as a diagnostic biomarker distinguishing between ischemic and hemorrhagic stroke. Furthermore, and importantly, we have shown that CCL5 levels at admission predict clinical outcomes in ischemic stroke patients, as measured by infarct volume growth at day 7. All these results identify CCL5 as a neuroprotective chemokine in stroke. The development of rapid tests for evaluating CCL5 levels may be useful for predicting patient outcomes, developing personalized treatments, and stratifying stroke patients in clinical trials.

## Figures and Tables

**Table 1:** Clinical characteristics of the 36 ischemic patients included in the study.

**Age (Years), Median (Q1–Q3)**		75 (63.5–79)
**Sex (M)**		20 (55.5%)
**Risk factors**		
	**Arterial hypertension**	25 (69.4%)
	**Diabetes mellitus**	12 (33.3%)
	**Dyslipidemia**	20 (55.5%)
	**Atrial fibrillation**	6 (16.6%)
	**Previous stroke**	7 (19.4%)
**Previous treatment**		
	**Antihypertensives**	22 (61.1%)
	**Statins**	14 (38.8%)
	**Antiaggregants**	14 (38.8%)
	**Anticoagulants**	9 (25.0%)
**TOAST**		
	**Atherothrombotic**	11 (30.5%)
	**Cardioembolic**	16 (44.4%)
	**Lacunar**	2 (5.5%)
	**Cryptogenic**	5 (13.8%)
	**Undetermined etiology**	2 (5.5%)
**mRankin**		
Basal	**0–1–2**	32 (88.8%)
	**3–4**	4 (11.1%)
	**5–6**	0 (0.0%)
3 months	**0–1–2**	21 (58.3%)
	**3–4**	10 (27.7%)
	**5–6**	5 (13.8%)
**NIHSS, median (Q1–Q3)**		
Basal		6 (2–13.2)
Hospital discharge		1 (0–8)
3 months		0 (0–5.2)
**Infarct volume at d0 (cm^3^), median (Q1–Q3)**		4.9 (1.3–22.6)
**Infarct volume at d7 (cm^3^), median (Q1–Q3)**		3.2 (1.4–20.8)
**CCL5 at d0 (ng/mL), median (Q1–Q3)**		42.3 (24.3–50.6)
**CCL5 at d1 (ng/mL), median (Q1–Q3)**		43.1 (25.6–56.4)
**CCL5 at HD (ng/mL), median (Q1–Q3)**		60.4 (35.4–71.9)

**Table 2:** Clinical characteristics of the 64 hemorrhagic patients included in the study.

**Age (Years), Median (Q1–Q3)**		81 (72.7–87)
**Sex (M)**		38 (59.4%)
**Risk factors**		
	**Arterial hypertension**	49 (76.6%)
	**Diabetes mellitus**	15 (23.4%)
	**Dyslipidemia**	20 (31.2%)
	**Atrial fibrillation**	18 (28.1%)
**Previous treatment**		
	**Antihypertensives**	45 (70.3%)
	**Statins**	21 (32.8%)
	**Antiaggregants**	19 (29.7%)
	**Anticoagulants**	20 (31.5%)
**TOAST**		
	**Supratentorial**	41 (64.1%)
	**Infratentorial**	6 (9.4%)
	**Lobar**	14 (21.9%)
	**Mixed**	3 (4.7%)
**mRankin**		
Basal	**0–1–2**	54 (84.4%)
	**3–4**	10 (15.6%)
	**5–6**	0 (0.0%)
3 months	**0–1–2**	28 (43.7%)
	**3–4**	12 (18.7%)
	**5–6**	24 (37.5%)
**NIHSS, median (Q1–Q3)**		
Basal		7 (2–16)
Hospital discharge		2.5 (1–6.2)
3 months		1.5 (0–3)
**Hematoma at d0 (cm^3^), median (Q1–Q3)**		4.5 (1–13.9)
**Hematoma at d1 (cm^3^), median (Q1–Q3)**		4.1 (1–10.9)
**CCL5 at d0 (ng/mL), median (Q1–Q3)**		29.7 (18.2–45.6)
**CCL5 at d1 (ng/mL), median (Q1–Q3)**		28.9 (22.5–40.5)
**CCL5 at HD (ng/mL), median (Q1–Q3)**		33.9 (22.0–51.1)

## Data Availability

The datasets used and/or analyzed during the current study are available from the corresponding author on reasonable request.

## Supplementary Materials

The following supporting information can be downloaded at: https://www.mdpi.com/article/10.3390/ijms23179967/s1.

## Abbreviations

CCL5: chemokine (C-C motif) ligand 5; CV: coefficient of variation; GFAP: glial fibrillary acidic protein; HD: hospital discharge; mRankin: modified ranking scale; NIHSS: National Institutes of Health Stroke Scale; NMR: nuclear magnetic resonance; NT-proBNP: N-terminal prohormone of brain natriuretic peptide; RBP-4: retinol binding protein 4; SEM: standard error of the mean; TOAST: Trial of ORG 10172 in Acute Stroke Treatment.
