# Supplementary material for: CCL5 Levels Predict Stroke Volume Growth in Acute Ischemic Stroke and Significantly Diminish in Hemorrhagic Stroke Patients
Source: Int J Mol Sci. 2022 Sep 1;23(17):9967. doi: 10.3390/ijms23179967 (PMC9456070; doi:10.3390/ijms23179967)
Supplement: Supplementary file 1 [file ijms-23-09967-s001.zip › ijms-1846077-supplementary.pdf]

CCL5 Levels Predict Stroke Volume Growth in Acute Ischemic Stroke and Significantly Diminish in Hemorrhagic Stroke Patients

Francisco José Julián-Villaverde [1], Marta Serrano-Ponz [2], Enrique Ramalle-Gómara [3], Alfredo Martínez [4,†] and Laura Ochoa-Callejero [4,5,*,†]

1 Stroke Unit, Neurology Service, Hospital San Pedro, 26006 Logroño, Spain
2 Neurology Service, Hospital Universitario Miguel Servet, 50009 Zaragoza, Spain
3 Department of Epidemiology, La Rioja Government, 26071 Logroño, Spain
4 Angiogenesis Group, Oncology Area, Center for Biomedical Research of La Rioja (CIBIR), 26006 Logroño, Spain
5 Department of Nursing, University of La Rioja, 26004 Logroño, Spain
* Correspondence: locallejero@riojasalud.es; Tel.: +34-941278775; Fax: +34-941278887
† These authors contributed equally to this work.

Supplementary Materials

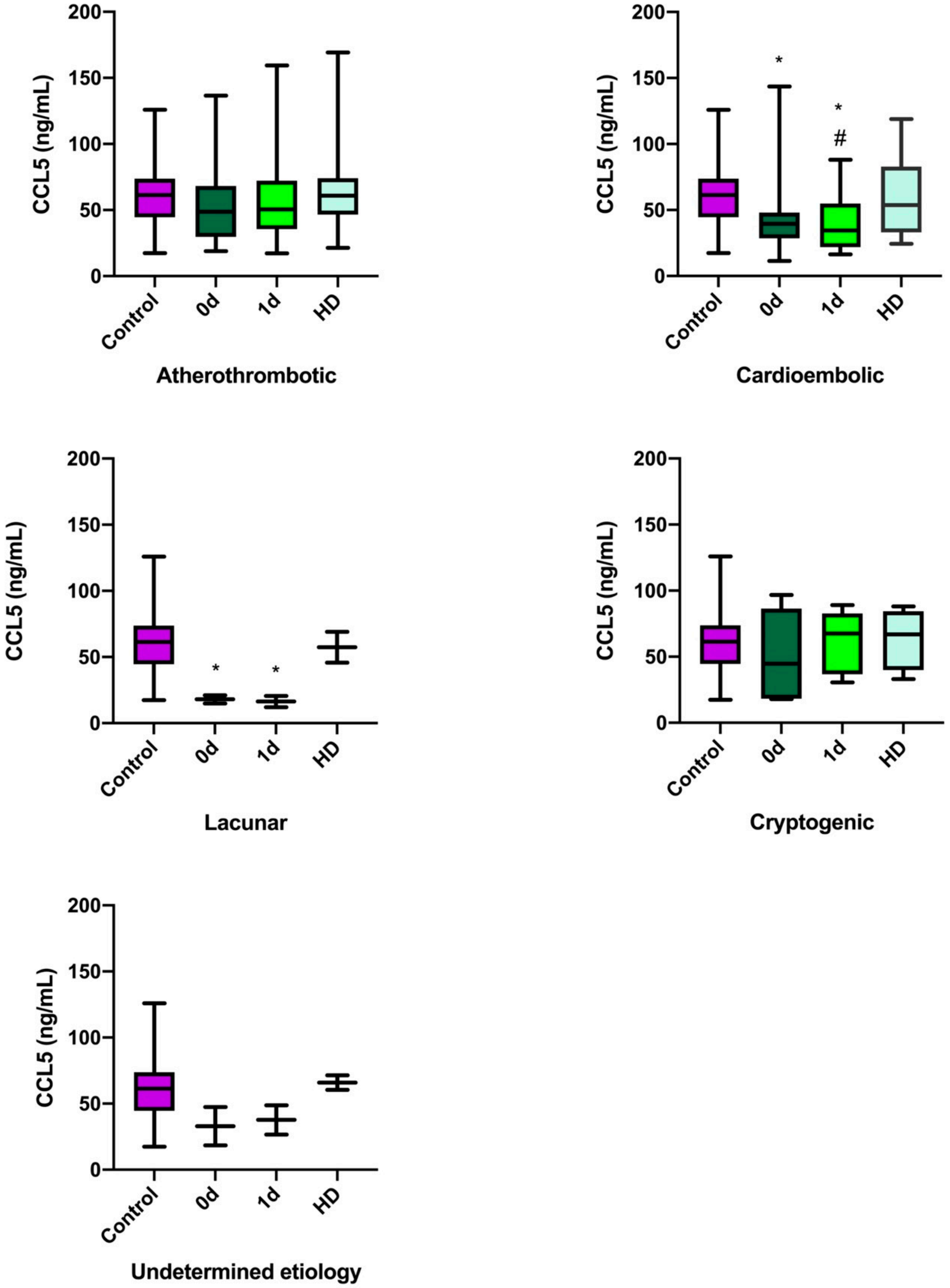

Figure S1. Etiologic classification of ischemic stroke. CCL5 levels in control and ischemic patients taken at 0 days, 1 day, and hospital discharge (HD) according to the different TOAST subtypes: Atherothrombotic (n=11), Cardioembolic (n=16), Lacunar (n=2), Cryptogenic (n=5), and Undetermined etiology (n=2). Box plots represent the interquartile range with the median as a horizontal line. Whiskers encompass the maximum and minimum values of the population. *: $p<0.05$ vs Control; and #: $p<0.05$ vs HD.

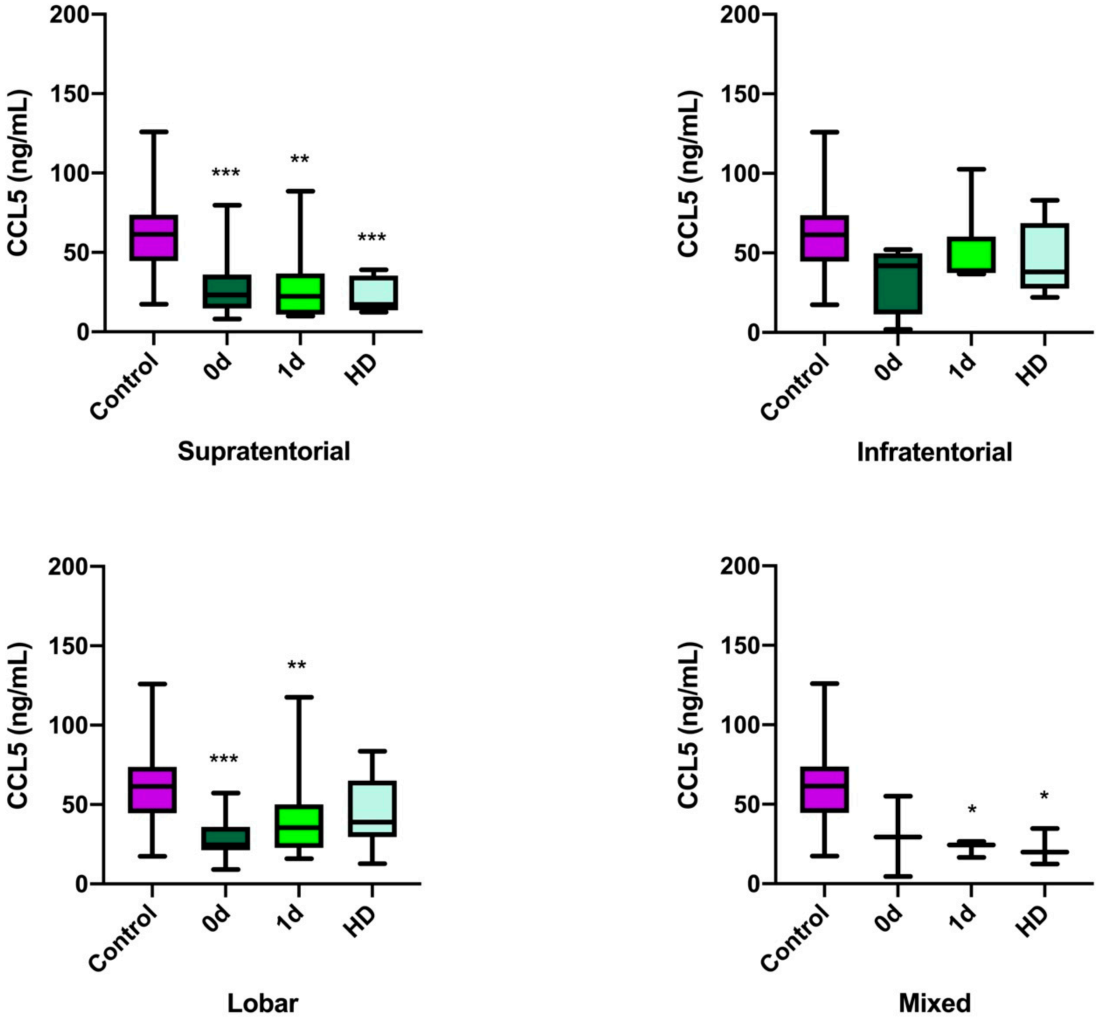

Figure S2. Etiologic classification of hemorrhagic stroke. CCL5 levels in control and hemorrhagic patients taken at 0 days, 1 day, and HD according to the different TOAST subtypes: Supratentorial (n=39), Infratentorial (n=8), Lobar (n=14), and Mixed (n=3). Box plots represent the interquartile range with the median as a horizontal line. Whiskers encompass the maximum and minimum values of the population. *: p<0.05; **: p<0.01, ***: p<0.001 vs Control.

A

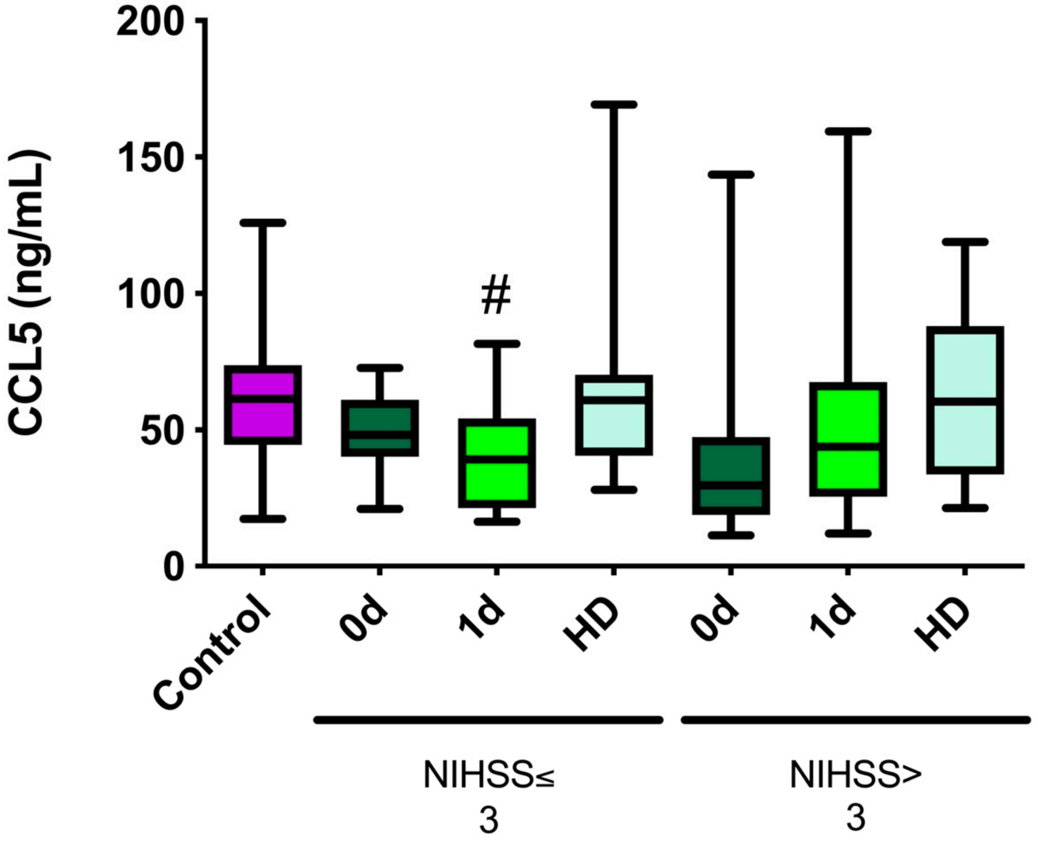
CCL5 (ng/mL)
200
150
100
50
0
#
Control
0d
1d
HD
0d
1d
HD
NIHSS≤
3
NIHSS>
3

B

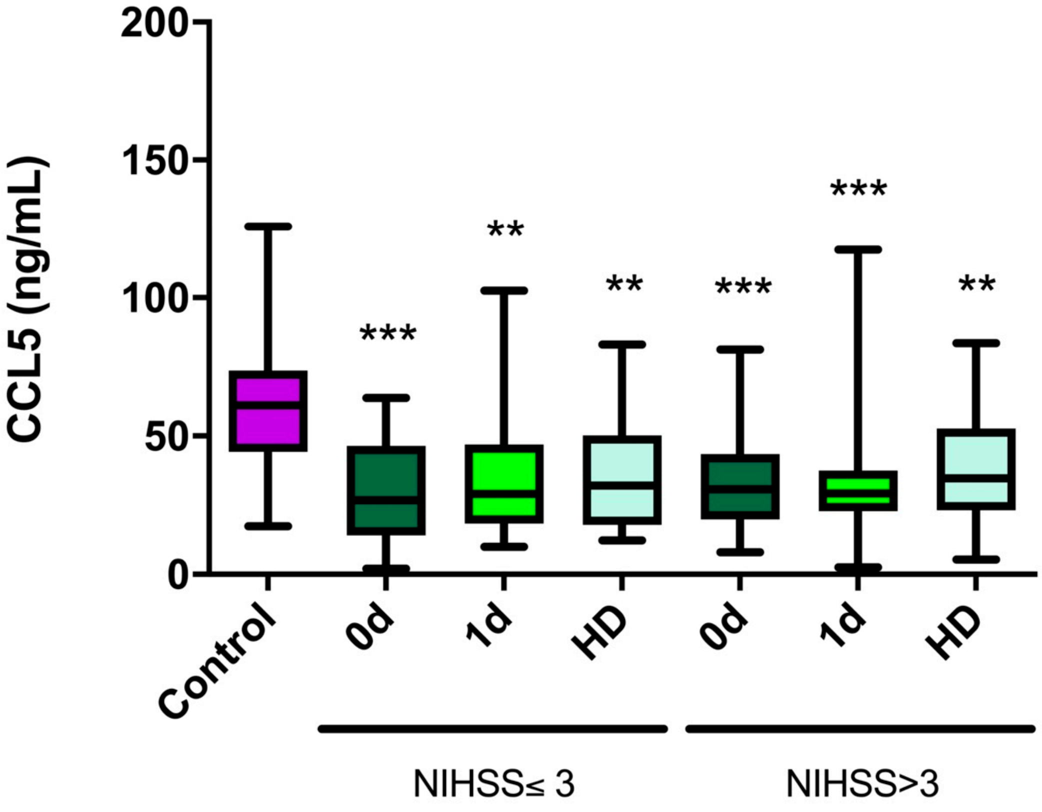
CCL5 (ng/mL)
200
150
100
50
0
***
**
**
***
***
**
Control
0d
1d
HD
0d
1d
HD
NIHSS≤ 3
NIHSS>3

Figure S3. CCL5 levels and their association with the NIHSS score at admission. The amount of circulating CCL5 was measured in ischemic (A) and hemorrhagic (B) stroke patients at days 0, 1, and HD. For each pathology, patients were assigned to two subgroups defined according to their NIHSS at admission; good prognosis (NIHSS≤3) or bad prognosis (NIHSS>3). Ischemic patients with good prognosis (n=13) had lower CCL5 levels at 1d as compared to HD. Patients with bad prognosis (n=23) had no differences in CCL5 levels than controls. Hemorrhagic patients were also divided into good prognosis (n=22) and bad prognosis (n=42) subgroups. All hemorrhagic patients showed lower CCL5 levels at every time point in comparison with controls (healthy volunteers). No differences were found among groups of hemorrhagic patients. Box plots represent the interquartile range with the median as a horizontal line. Whiskers encompass the maximum and minimum values of the population. **: $p<0.01$; ***: $p<0.001$; vs Control; and $^{\#}$: $p<0.05$ vs HD.

A

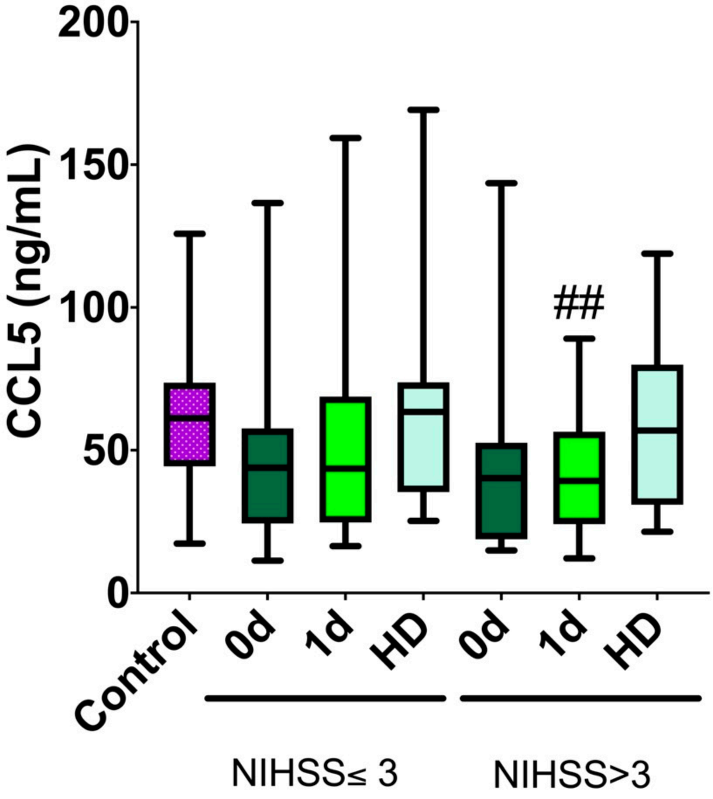

200
150
100
50
0
CCL5 (ng/mL)
##
Control
0d
1d
HD
0d
1d
HD
NIHSS≤ 3
NIHSS>3

B

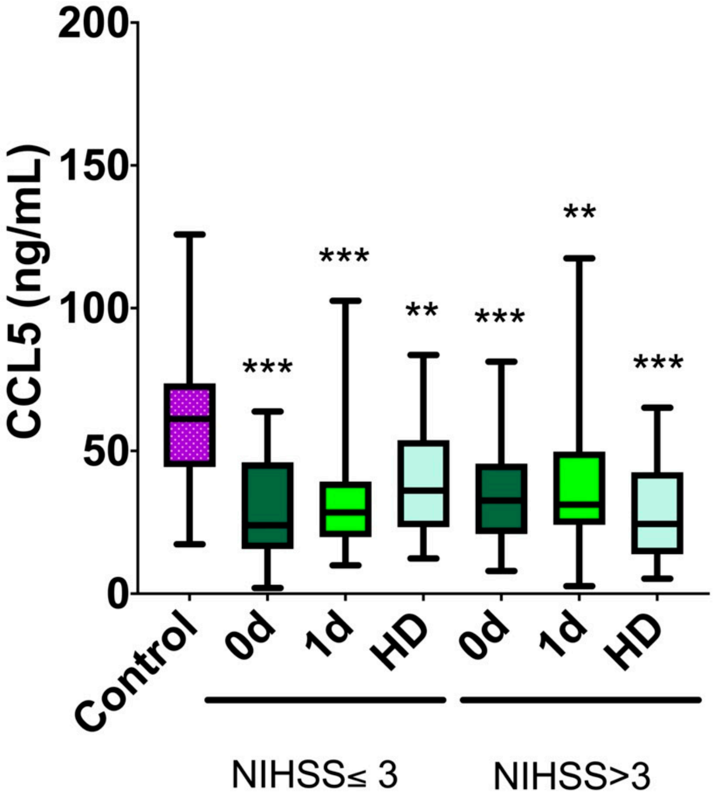

200
150
100
50
0
CCL5 (ng/mL)
***
***
**
***
**
***
Control
0d
1d
HD
0d
1d
HD
NIHSS≤ 3
NIHSS>3

Figure S4. CCL5 levels and their relationship with the NIHSS score 3 months after stroke. Patients were assigned to two subgroups defined according to their NIHSS at 3 months (A,B); good prognosis (NIHSS≤3, n=22 ischemic, n=33 hemorrhagic) or bad prognosis (NIHSS>3, n=14 ischemic, n=31 hemorrhagic). Ischemic patients with bad prognosis had lower CCL5 levels at 1d as compared to HD. All hemorrhagic patients showed lower CCL5 levels at every time point in comparison with controls (healthy volunteers). No differences were found among groups of hemorrhagic patients. No differences were found between good and bad prognosis in either stroke group. Box plots represent the interquartile range with the median as a horizontal line. Whiskers encompass the maximum and minimum values of the population.**: $p<0.01$; ***: $p<0.001$; vs Control. $^{\#\#}$ $p<0.01$ vs HD.
